# Oracles and Query Lower Bounds in Generalised Probabilistic Theories

**DOI:** 10.1007/s10701-018-0198-4

**Published:** 2018-07-12

**Authors:** Howard Barnum, Ciarán M. Lee, John H. Selby

**Affiliations:** 1 Department of Mathematical Sciences, University of Copenhagen, Copenhagen, Denmark; 2 Department of Physics and Astronomy, University of New Mexico, Albuquerque, USA; 3 Department of Physics and Astronomy, University College London, London, UK; 4 Department of Computer Science, University of Oxford, Oxford, OX1 3QD UK; 5 Imperial College London, London, SW7 2AZ UK

**Keywords:** Generalised probabilistic theories, Query complexity, Oracles, Higher order interference

## Abstract

We investigate the connection between interference and computational power within the operationally defined framework of generalised probabilistic theories. To compare the computational abilities of different theories within this framework we show that any theory satisfying four natural physical principles possess a well-defined oracle model. Indeed, we prove a subroutine theorem for oracles in such theories which is a necessary condition for the oracle model to be well-defined. The four principles are: causality (roughly, no signalling from the future), purification (each mixed state arises as the marginal of a pure state of a larger system), strong symmetry (existence of a rich set of nontrivial reversible transformations), and informationally consistent composition (roughly: the information capacity of a composite system is the sum of the capacities of its constituent subsystems). Sorkin has defined a hierarchy of conceivable interference behaviours, where the order in the hierarchy corresponds to the number of paths that have an irreducible interaction in a multi-slit experiment. Given our oracle model, we show that if a classical computer requires at least *n* queries to solve a learning problem, because fewer queries provide no information about the solution, then the corresponding “no-information” lower bound in theories lying at the *k*th level of Sorkin’s hierarchy is $\lceil {n/k}\rceil$. This lower bound leaves open the possibility that quantum oracles are less powerful than general probabilistic oracles, although it is not known whether the lower bound is achievable in general. Hence searches for higher-order interference are not only foundationally motivated, but constitute a search for a computational resource that might have power beyond that offered by quantum computation.

Landauer’s Principle [1] states that any logically irreversible processing or manipulation of information, such as the erasure of a bit, must always be accompanied by an entropy increase in the environment of the system processing the information. As this illustrates, information is intimately tied to the physical system that embodies it and is hence bound by physical law—alternatively, *information is physical*. If information processing—or computation—is bound by physical law, then the ultimate limits of computation should be derivable from natural physical principles. Indeed, the advent of quantum computation demonstrated that different physical principles lead to different limits on computational power. This naturally leads to the question of what general relationships hold between computational power and physical principles. This question has recently been studied in the framework of generalised probabilistic theories [2–7], which contains operationally-defined physical theories that generalise the probabilistic formalism of quantum theory. By studying how computational power varies as the underlying physical theory is changed, one can determine the connection between physical principles and computational power in a manner not tied to the specific mathematical manifestation of a particular principle within a theory.

Most previous research into computation within the generalised probabilistic theory framework has focused on deriving general *limitations* on computational ability from natural physical principles.^1^ No work to date has tied a computational *advantage* directly to a physical principle. For instance, it is known that quantum interference between computational paths is a resource for post-classical computation [8], but it is not clear whether the presence of interference in a general theory entails post-classical computation [9], nor whether post-quantum interference behaviour is in general a resource for post-quantum computation [5]. The former point concerns whether it is just the particular mathematical description of interference in Hilbert space which can be exploited to provide an advantage over classical computation or whether such a statement can be seen to directly follow from the observation of interference in nature, and the latter concerns whether “more” interference implies, or at least can sometimes allow, “more” computational power.

Indeed, as first noted by Sorkin [10, 11], there is a limit to quantum interference—at most *pairs* of computational paths can ever interact in a fundamental way. Sorkin has defined a hierarchy of operationally conceivable interference behaviours—currently under experimental investigation [12–15]—where classical theory is at the first level of the hierarchy and quantum theory belongs to the second. Informally, the order in the hierarchy corresponds to the number of paths that have an irreducible interaction in a multi-slit experiment. Given this definition, one can investigate the role interference plays in computation in a theory-independent manner by asking whether theories at level *k* possess a computational advantage over theories at level $k-1, k-2, \ldots$.

One usually demonstrates the existence of a quantum advantage over classical computation using *oracles*. Indeed, the Deutsch-Jozsa problem [16] is such a example: given a function $f:\{0,1\}^n\rightarrow \{0,1\}$, one is asked to determine whether it is constant (same output for all inputs) or balanced (0 on exactly half of the inputs)—promised that it is one of these cases. Performance is quantified by the number of queries to an oracle, which implements *f* on any desired input, needed to solve the problem. A deterministic classical computer requires $2^{n-1}+1$ queries, but Deutsch and Jozsa showed that a quantum computer can solve the problem in a single query to an appropriately defined *quantum* oracle. Hence, to compare the computational abilities of different theories within the framework a well-defined oracle model is needed. We show that such a model can be defined in any theory satisfying the following physical principles: *causality* (roughly, no signalling from the future), *purification* (each mixed state arises as the marginal of a pure state of a larger system), and *strong symmetry* (existence of a rich group of non-trivial reversible transformations); additionally, we demand *informationally consistent composition* (the act of bringing systems together cannot create or destroy information). Moreover, we prove a subroutine theorem for theories satisfying these principles. That is, we show that having access to an oracle for a particular decision problem which can be efficiently solved in a given theory does not provide any more computational power than just using the efficient algorithm itself. Such a result was proved for quantum theory by Bennett et al. [17], and is a necessary condition for a well-defined oracle model.

Given this oracle model, we investigate whether lower bounds on the number of queries needed by a quantum computer to solve certain computational problems can be reduced in theories which possess higher-order interference and satisfy the principles discussed above. Indeed, we generalise results due to Meyer and Pommersheim [18] and show that if a particular type of lower bound to a given query problem (from a fairly large class of such problems) is *n* using a classical computer, then the corresponding lower bound for the same problem in theories with *k*th order interference is $\lceil {n/k}\rceil$. As quantum theory only exhibits second order interference, theories with post-quantum interference allow for post-quantum computation. For example, in the problem where we are asked to determine the parity of a function $f:\{1,\ldots ,k\}\rightarrow \{0,1\}$ (which generalizes the special case of the Deutsch-Jozsa problem for functions $f:\{0,1\} \rightarrow \{0,1\}$), $\lceil {k/2}\rceil$ quantum queries are needed, but we show that in any theory satisfying our principles which has *k*th order interference, our generalisation of the Meyer-Pommersheim useless-queries bound leaves open the possibility that the parity can be determined with a single query. This bound leaves open the possibility that the power of computation might be improved by modifying interference behaviour in an operationally conceivable manner. Hence searches for higher-order interference are not only foundationally motivated, but constitute a search for a computational resource potentially beyond that offered by quantum computation. An important direction for future work is to determine whether in theories satisfying these principles it is possible to find an algorithm that reaches this lower bound. We discuss potential ways to do this in the conclusion.

Other authors have considered computation beyond the usual quantum formalism from a different perspective to the one employed here. For example, Aaronson has considered alternate modifications of quantum theory, such as a hidden variable model in which the history of hidden states can be read out by the observer, and—together with collaborators in [19]—a model in which one is given the ability to perform certain unphysical non-collapsing measurements. Both of these models have been shown to entail computational speed-ups over the usual quantum formalism. Additionally, Bao et al. [20] have investigated computation in modifications of quantum theory suggested by the black hole information loss paradox and have shown the ability to signal faster than light in such theories is intimately linked to a speed-up over standard quantum theory in searching an unstructured database. In contrast, the generalised probabilistic theory framework employed here allowed for an investigation of query lower bounds and computational advantages in alternate theories that are physically reasonable and which, for instance, do not allow for superluminal signalling [21], cloning [22], or other phenomena that are arguably undesirable features of a theory.

The paper is organised as follows. In Sect. 1, the generalised probabilistic theory framework will be introduced along with our physical principles and the definition of higher-order interference. In Sect. 2 the oracle model will be introduced and defined and the subroutine theorem will be stated, with the proof presented in Appendix C. Finally, in Sect. 3 we derive lower bounds on query problems that directly follow from our principles.

## The Framework

One of the fundamental requirements of a physical theory is that it should provide a consistent account of experimental observations. This viewpoint underlies the framework of generalised probabilistic theories [21, 23–25]. A generalised probabilistic theory specifies a set of laboratory devices that can be connected together in different ways to form experiments and specifies probability distributions over experimental outcomes. A device comes equipped with input ports, output ports, and a classical pointer. When a device is used in an experiment, the classical pointer comes to rest in one of a number of positions (“values”), indicating an outcome has occurred. Intuitively, one envisages *physical systems* passing between the ports of these devices. Such physical systems come in different types, denoted $A,B,\ldots$. One constructs experiments by composing devices both sequentially and in parallel, and when composed sequentially, types must match.

In this framework, closed circuits—those with no unconnected ports and no cycles—are associated with probabilities—a probability for each assigment of values to all classical pointers appearing in the circuit. These add to unity when summed over all assignments of values to pointers in the circuit. We use the term “element” for a pair of device and pointer value; circuits may also be constructed of such elements, and closed circuits of this type are associated with individual probabilities. The set of equivalence classes of elements with no input ports are called *states*, no output ports *effects* and both input and output ports *transformations*. The set of all states of system *A* is denoted $\mathrm {St(A)}$, the set of all effects on *B* is denoted $\mathrm {Eff(B)}$ and the set of transformations between systems *A* and *B* is denoted $\mathrm {Transf(A,B)}$. Using standard operational assumptions and arguments [3, 21, 23], one can show that the set of states, effects and transformations each give rise to a real vector space with transformations and effects acting linearly on the real vector space of states. We assume in this work that all vector spaces are finite dimensional.^2^

A state is said to be *pure* if it does not arise as a *coarse-graining* of other states^3^; a pure state is one for which we have maximal information. A state is *mixed* if it is not pure. A state $\omega$ is *completely mixed* if for every pure state $\chi$, $\omega$ can be expressed as a coarsegraining of a set of states that includes $\chi$. Similarly, one says a transformation, respectively an effect, is pure if it does not arise as a coarse-graining of other transformations, respectively effects. It can be shown that reversible transformations preserve pure states [26]. We’ll say a measurement is pure if all of its outcomes are pure effects. A state is *maximally mixed* if, when expressed as a convex combination $\omega = \sum _{i \in S} p_i \omega _i$ of perfectly distinguishable pure states, as is always possible given our assumptions, the probabilities $p_i$ are uniform ($p_i = 1/|S|$).

The following ‘Dirac-like’ notation $$_A|s_i)$$ will be used to represent a state^4^ of system *A*, and $$(e_{r}|_B$$ to represent an effect on *B*. Here *i* and *r* represent the position of the classical pointer associated to the device the prepares the state and performs the measurement, respectively. The full measurement is defined by the collection $$\{(e_r|\}_r$$. States, effects, and transformations can be represented diagrammatically:The above diagram represents the joint probability of preparing state $$|s_i)$$, acting with transformation *T* and registering outcome *r* for the measurement $$\{(e_r|\}_r$$. In the above, the wires represent physical systems, with their type denoted by the letter above them. This diagrammatic representation was inspired by categorical quantum mechanics [27, 28]. Note that in the “bra-ket” like notation, the time-ordering (first states, then transformations, then effects) “flows” from right to left, while the reverse is true in the diagrammatic notation.

In the rest of the paper, it will be assumed that all theories satisfy the following physical principles.

### Definition 1.1

(*Deterministic effect, Causality* [23]) An effect is called *deterministic* if it has probability 1 in all states. A theory is said to be *causal* if there exists a unique deterministic effect for every system. We denote this effect by . In a causal theory,  for all measurements $\{(e_r|\}_r$ on a given system.

Mathematically, the principle of causality is equivalent to the statement: “Probabilities of outcomes of present experiments are independent of future measurement choices”. In causal theories, all states are *normalised* [23]. That is,  for all |*s*). Moreover, the unique deterministic effect allows one to define a notion of *marginalisation* for multi-partite states.

### Definition 1.2

(*Purification* [23]) *Given a state* $$_A|s)$$ *there exists a systemB* *and a pure state*$$_{AB}|\psi )$$ on *AB* such that $$_A|s)$$ is the marginalisation of $$_{AB}|\psi )$$:Moreover, the purification $$_{AB}|\psi )$$ is unique up to reversible transformations on the purifying system, *B*. That is if two states $$_{AB}|\psi )$$ and $$_{AB}|\psi ')$$ purify $$_A|s)$$, then there exists a reversible transformation $$T_B$$ on system *B* such that $$_{AB}|\psi )=(\mathbb {I}_A\otimes {T_B})~_{AB}|\psi )$$.

As pure states are those in which we have maximal information about a system^5^ purification principle formalises the statement that each state of incomplete information about a system can arise in an essentially unique way due to a lack of full access to a larger system that it is part of. In [29] it was argued that purification can be thought of as a statement of “information conservation”. In a theory with purification, any missing information about the state of a given system can always be traced back to lack of access to some environment system.

We introduce one final principle which ensures that the information stored in a system is compatible with composition, that is, we demand that the mere act of bringing systems together should not create or destroy information.^6^ If this were not the case then one could potentially use this new global degree of freedom, representing the increase of information capacity, to hide solutions to a hard computational problem allowing one to solve a hard problem that could not be solved by using the systems independently [3]. We formalise this as follows:

### Definition 1.3

(*Informationally consistent composition*) This consists of two constraints on parallel composition: (i) the product of pure states is pure, (ii) the product of maximally mixed states is maximally mixed.

The first of these formalises the intuitive idea that if one has maximal information about each of two systems, then one has maximal information about the composite of the two systems. The existence of a maximally mixed state, that is, a state about which we have minimal information, is guaranteed for each system by purification [23].

The purification principle, in conjunction with causality and the constraints on parallel composition discussed above, implies many quantum information processing [23] and computational primitives [5]. Examples include teleportation, no information without disturbance, and no-bit commitment [23]. Moreover, purification also leads to a well-defined notion of thermodynamics [26, 30, 31]. Quantum theory—both on complex and real Hilbert spaces—satisfies purification as do Spekkens’ toy model [32, 33] purification distinct from quantum theory include fermionic quantum theory [34, 35], a superselected version of quantum theory known as double quantum theory [31], and a recent extension of classical theory to the theory of coherent *d*-level systems, or codits [26].

Pure states $\{|s_i)\}_{i=1}^n$ are called *perfectly distinguishable* if there exists a measurement, corresponding to effects $\{(e_j|\}_{j=1}^n$, with the property that $(e_j|s_i)=\delta _{ij}$ for all *i*, *j*.

### Definition 1.4

(*Strong symmetry* [36, 37]) A theory satisfies *strong symmetry* if, for any two *n*-tuples of pure and perfectly distinguishable states $\{|\rho _i)\},\{|\sigma _i)\},$ there exists a reversible transformation *T* such that $T|\rho _i)=|\sigma _i)$ for $i=1,\dots ,n$.

Although complex and real quantum theory satisfy all of the above principles, double quantum theory and codit theory do not satisfy strong symmetry. Whether any other theories satisfy all of the principles is an important open question.

The following consequences of the above principles, proved in [9], will be required to define oracles in Sect. 2.

### Definition 1.5

Given a set of pure and perfectly distinguishable states $\{|i)\}$, and a set of transformations $\{T_i\}$, define a controlled transformation $C\{T_i\}$ as one that satisfies:1.1The top system and lower systems are referred to as the *control* and *target* respectively.

Note that classically-controlled transformations—those in which the control is measured and, conditioned on the outcome, a transformation is applied to the target—exist in any causal theory with sufficiently many distinguishable states [23]. However, such transformations are in general not reversible [9].

### Theorem 1.6

([9] Theorem 2) In any theory satisfying (i) causality, (ii) purification, (iii) strong symmetry, (iv) product of pure states is pure, and in which there exists a set of *n* pure and perfectly distinguishable states |*i*) indexed by $i \in \{1,\ldots ,n\}$, for any collection of *n* reversible transformations $\{T_i\}_{i=1}^n$ there exists a *reversible* controlled transformation $C\{T_i\}$ in which the $T_i$ are controlled by the states |*i*).

Every controlled unitary transformation in quantum theory has a *phase kick-back* mechanism [16, 38]. Such mechanisms form a vital component of most quantum algorithms [38]. It was shown in [9] that a *generalised* phase kick-back mechanism exists in any theory satisfying the above physical principles.

### Definition 1.7

A *phase transformation*, relative to a given measurement of effects (*i*|, is a transformation *Q* such that:


### Theorem 1.8

([9] Lemma 2) In a theory satisfying Causality, Strong Symmetry, and Purification, for any set $$T_i$$ of reversible transformations and state |*s*) such that for all *i* $$T_i|s)=|s)$$, there exists a reversible transformation *Q* such that1.2where *Q* is a phase transformation. Moreover, every phase transformation can arise via such a generalised phase kick-back mechanism.

### Post-quantum Interference

In the sections that follow, we will connect post-quantum, or higher-order, probabilistic interference to post-quantum computation; investigating whether “more” interference implies “more” computational power. The definition of higher-order interference that we present here takes its motivation from the set-up of multi-slit interference experiments. In such experiments a particle (a photon or electron, say) passes through slits in a physical barrier and is detected at a screen placed behind the barrier. By blocking some (or none) of the slits and repeating the experiment many times, one can build up an interference pattern on the screen. The “intensity” of the pattern in a small area of the screen is proportional to the probability that the particle arrives there. Informally, a theory has “*n*th order interference” if one can generate interference patterns in an *n*-slit experiment which cannot be created in any experiment with only *m* slits, for all $m<n$.

More precisely, this means that the interference pattern created on the screen cannot be written as a particular linear combination of the interference patterns generated when different subsets of slits are open and closed. In the standard two slit experiment, quantum interference corresponds to the statement that the interference pattern can’t be written as the sum of single slit patterns:It was first shown by Sorkin [10, 11] that—at least for ideal experiments [39]—quantum theory is limited to the $n=2$ case. That is, the interference pattern created in a three—or more—slit experiment *can* be written in terms of the two and one slit interference patterns obtained by blocking some of the slits. Schematically:The terms with minus signs in the above correct for over-counting of the open slits. If a theory does not have *n*th order interference then one can show it will not have *m*th order interference, for any $m>n$ [10]. Therefore, one can classify theories according to their maximal order of interference, *k*. For example quantum theory lies at $k=2$ and classical theory at $k=1$.

Consider the state of the particle just before it passes through the slits. For every slit, there should exist states such that the particle would definitely be found at that slit, if one were to measure it. Mathematically, this means that there exists a face^7^ [36] of the state space, such that all states in this face give unit probability for the “yes” outcome of the two outcome “is the particle at this slit?” measurement. These faces will be labelled $F_i$, one for each of the *n* slits $i\in \{1,\ldots , n\}$. As the slits should be perfectly distinguishable, the faces associated to the slit should be mutually orthogonal. This can be achieved by letting the slits be in one-to-one correspondence with a set of pure and perfectly distinguishable states.

One can additionally ask coarse grained questions of the form “Is the particle found among a certain subset of slits, rather than somewhere else?”. The set of states that give outcome “yes” with probability one must contain all the faces associated with each slit in the subset. Hence the face associated to the subset of slits $I\subseteq \{1,\ldots , n\}$ is the smallest face containing each face in this subset, $F_I:=\bigvee _{i\in I} F_i$. That is, $F_I$ is the face generated by the pure and perfectly distinguishable states identified by the subset *I*. The face $F_I$ contains all those states which can be found among the *I* slits. The experiment is “complete” if all states in the state space (of a given type *A*) can be found among some subset of slits. That is, if $F_{12\cdots n}=\mathrm {St(A)}$.

Higher-order interference was initially formalised by Rafael Sorkin in the framework of Quantum Measure Theory [10] but has more recently been adapted to the setting of generalised probabilistic theories in [9, 36, 40–42]. A straightforward translation to this setting describes the order of interference in terms of probability distributions corresponding to interference patterns generated in the different experimental setups (which slits are open, etc.) [9, 42]. However, given the principles imposed in the previous section, it is possible to define physical transformations that correspond to the action of opening and closing certain subsets of slits. In this case, there is a more convenient (and equivalent [36], given our principles) definition in terms of such transformations (such a definition was also used in [40, 41]).

Given *N* slits, labelled $1, \ldots , N$, these transformations will be denoted $P_I$, where $I \subseteq \{1, \ldots , N\}$ corresponds to the subset of slits which are not closed. In general one expects that $P_I P_J = P_{I\cap J}$, as only those slits belonging to both *I* and *J* will not be closed by either $P_I$ or $P_J$. This intuition suggests that these transformations should correspond to projectors (i.e., idempotent transformations $P_IP_I=P_I$). Given the principles imposed in this paper, this is indeed the case.

#### Theorem 1.9

In any theory satisfying the principles introduced in the previous section, the projector onto a face generated by a subset of pure and perfectly distinguishable states is an allowed transformation in the theory. If *F* and *G* are faces generated by subsets of the same pure and perfectly distinguishable set of states, one has $P_FP_G=P_{F\cap G}$.

The proof of theorem 1.9 is presented in Appendix A. Given this structure, one can define the maximal order of interference as follows [5, 36].

#### Definition 1.10

A theory satisfying the principles imposed in this section has maximal order of interference *k* if, for any $N \ge k$, one has:

$$\begin{aligned} \mathbb {1}_N = \sum _{{ \begin{array}{c} I\subseteq \mathbf {N} \\ |I|\le k \end{array}}}\mathcal {C}\left( k,|I|,N\right) P_I \end{aligned}$$

where $\mathbb {1}_N$ is the identity on a system with *N* pure and perfectly distinguishable states and

$$\begin{aligned} \mathcal {C}\left( k,|I|,N\right) :=(-1)^{k-|I|}\left( \begin{array}{c}N-|I|-1\\ k-|I|\end{array}\right) \end{aligned}$$

The factor $\mathcal {C}\left( k,|I|,N\right)$ in the above definition corrects for the overlaps that occur when different combinations of slits are open and closed. For $k=N$, the above reduces to the expected expression $\mathbb {1}_h=P_{\{1,\ldots ,k \}}$, that is, the identity is given by the projector with all slits open. The case $N=k+1$ corresponds to $\mathcal {C}\left( k,|I|,k+1\right) = (-1)^{k-|I|}$, corresponding to the situation depicted in the above diagrams, as well as the one most commonly discussed in the literature [10, 40].

Instead of working directly with these physical projectors, it is mathematically convenient to work with the (generally) unphysical transformations corresponding to projecting onto the “coherences” of a state. Consider the example of a qutrit in quantum theory, the projector $P_{\{0,1\}}$ projects onto a two dimensional subspace:

$$\begin{aligned} P_{\{0,1\}}::\left( \begin{array}{ccc} \rho _{00} &{} \rho _{01} &{} \rho _{02} \\ \rho _{10} &{} \rho _{11} &{} \rho _{12} \\ \rho _{20} &{} \rho _{21} &{} \rho _{22} \end{array}\right) \mapsto \left( \begin{array}{ccc} \rho _{00} &{} \rho _{01} &{} 0 \\ \rho _{10} &{} \rho _{11} &{} 0 \\ 0 &{} 0 &{} 0 \end{array}\right) \end{aligned}$$

whilst the coherence-projector $\omega _{\{0,1\}}$ projects only onto the coherences in that two dimensional subspace:

$$\begin{aligned} \omega _{\{0,1\}}::\left( \begin{array}{ccc} \rho _{00} &{} \rho _{01} &{} \rho _{02} \\ \rho _{10} &{} \rho _{11} &{} \rho _{12} \\ \rho _{20} &{} \rho _{21} &{} \rho _{22} \end{array}\right) \mapsto \left( \begin{array}{ccc} 0 &{} \rho _{01} &{} 0 \\ \rho _{10} &{} 0 &{} 0 \\ 0 &{} 0 &{} 0 \end{array}\right) . \end{aligned}$$

That is, $\omega _{\{0,1\}}$ corresponds to the linear combination of projectors: $P_{\{0,1\}}-P_{\{0\}}-P_{\{1\}}$.

There is a coherence-projector $\omega _I$ for each subset of slits $I \subseteq \{1,\ldots , N\}$, defined in terms of the physical projectors:

$$\begin{aligned} \omega _I:=\sum _{\tilde{I}\subseteq I}(-1)^{|I|+|\tilde{I}|}P_{\tilde{I}}. \end{aligned}$$

These have the following useful properties, which were proved in [5, 36].

#### Lemma 1.11

An equivalent definition of the maximal order of interference, *k*, is:

$$\begin{aligned} \mathbb {1}_N=\sum _{I,|I|=1}^{k}\omega _I, \text { for all } N \ge k. \end{aligned}$$

The above lemma implies that any state (indeed, any vector in the vector space generated by the states) in a theory with maximal order of interference *k* can be decomposed in a form reminiscent of a rank *k* tensor:

1.3 $$\begin{aligned} |s)=\sum _{I,|I|=1}^k \omega _I|s)=\sum _{I,|I|=1}^k |s_I). \end{aligned}$$

This decomposition can be thought of as a generalised superposition, as it manifestly describes the coherences between different subsets of perfectly distinguishable states (the analogue of a basis in quantum theory) present in a given state. This will be important in discussing the power of oracle queries in the following section, since it allows oracles to be queried not just on states corresponding to definite inputs or probabilistic mixtures of them, but on superpositions of them.

## Oracles

In classical computation, an *oracle* is usually defined as a total function $f: S \rightarrow T$ from a finite or (more usually) countably infinite set *S* to a finite set *T*. Most commonly, we have $f:\mathbb {N}\rightarrow \{0,1\}$, or $f: \{0,1\}^* \rightarrow \{0,1 \}$. These last are essentially equivalent since the set $\{0,1\}^*$ of finite binary strings can be identified with $\mathbb {N}$ via the usual binary encoding. The string *x* is said to be *in* an oracle *O* if $f(x)=1$, hence oracles can decide membership in a language (defined as a subset of the set of finite binary strings). In a classical oracle model of computation, some baseline computational model, e.g. a circuit or Turing machine model, is augmented by the ability to “query” the oracle, i.e. obtain the value of *f* on one of its inputs. A query is assigned some cost in units commensurate with those of the baseline model, and multiple queries may be made in the course of a computation, on inputs provided in terms of the baseline model (normally, bit strings on a tape or in some set of registers). Oracle outputs are also provided in terms of the baseline model, and may be further processed by means of the baseline model’s resources and/or as input to additional queries. Sometimes a model is considered in which the resources of the baseline model are taken to be free, and the *only* cost is the number of queries to the oracle; this is usually termed a *query model*.

In quantum computation oracle queries to a function $f: \{0,1\}^* \rightarrow \{0,1\}$ are usually represented by a family $\{G_n\}$ of quantum gates, one for each length *n* of the “input” string *x*.^8^ Each $G_n$ is a unitary transformation acting on $n+1$ qubits, whose effect on the computational basis is in general given by

2.1 $$\begin{aligned} G_n|x,a\rangle = |x,a\oplus {f_n(x)}\rangle \end{aligned}$$

for all $x\in \{0,1\}^n$ and $a\in \{0,1\}$, where $f_n$ are a family of Boolean functions that represent the specific oracle under consideration. Since the family $f_n$ determines (and is determined by) a function $f: \{0,1\}^* \rightarrow \{0,1\}$, where $f_n$ is *f*’s restriction to inputs of length *n*, a quantum oracle may be thought of as a “coherent” version of the corresponding classical oracle *f*; we call it a “quantum oracle for *f*”. Slightly more generally, one could define a quantum oracle (“for *f*”) as a family (indexed by |*x*|) of controlled unitary transformations which, when queried by state $|x\rangle$ in the control register, applies a unitary—chosen from a set of two unitaries according to the value $f_n(x)$—to the target register. A specific example of a quantum oracle of this sort is the following controlled unitary:

2.2 $$\begin{aligned} U_f= |0\rangle \langle 0|\otimes Z^{f(0)} + |1\rangle \langle 1|\otimes Z^{f(1)}, \end{aligned}$$

with *Z* the Pauli *Z* matrix, $f:\{0,1\}\rightarrow \{0,1\}$ a function encoding some decision problem and $Z^{0}:=\mathbb {I}$. (If we had used the Pauli *X* matrix, in place of *Z*, this oracle would be of the type described by Eq. 2.1.

As was briefly mentioned in [9], the results of theorem 1.6 provide a way to define computational oracles in any theory satisfying our assumptions.

### Definition 2.1

In an theory satisfying our assumptions, an oracle for a decision problem $f: S \rightarrow T$, with *S* and *T* finite sets, is a reversible controlled transformation^9^ $C\{T_i\}$ where the set of transformations $\{T_i \}$ being controlled depend on $i \in S$ *only* through the value *f*(*i*). If $S = \{A\}^*$, the countably infinite set of strings from a finite alphabet *A*, then an oracle for *f* is defined to be a family $G_n$, indexed by the length *n* of strings, of such controlled transformations, one for each $f_n$, where $f_n$ is defined to be *f* restricted to strings of length *n*. If $T = \{0,1\}$, this is a decision problem.

In quantum theory there is an equivalent view of oracles in terms of phase transformations. This can be seen as a result of the phase kick-back algorithm [16, 38]. In the quantum example above, the phase kick-back for $U_f$ amounts to first rewriting $U_f$ as:

2.3 $$\begin{aligned} U_f=\mathbb {I}\otimes |0\rangle \langle 0|+Z^{f(0)\oplus f(1)}\otimes |1\rangle \langle 1|. \end{aligned}$$

Inputting $|1\rangle$ into the second qubit results in a ‘kicked-back’ phase of $Z^{f(0)\oplus f(1)}$ on the first qubit. The value of $f(0)\oplus f(1)$ can then be measured by preparing the first qubit in the state $|+\rangle$ and then measuring it in the $\{|+\rangle ,|-\rangle \}$ basis. This provides the value $f(0)\oplus f(1)$ in a single query of the oracle—a feat impossible on a classical computer [18].

In our more general setting, an analogue of the above holds via Theorem 1.8. That is, in theories satisfying our assumptions, as the transformations $T_{i}$ depend on the value *f*(*i*), so too can the controlled transformation and the kicked-back phase. That is, in theories with non-trivial phases, i.e. non-classical theories, the phase kick-back of an oracle can encode information about the value *f*(*i*) for all *i*. Indeed, it is also the case that if one has available as one circuit element the generalised phase kick-back transformation constructed out of the controlled transformation $C{T_i}$ one can construct a circuit for $C{T_i}$ [9]. Hence—as in the quantum example above—from the point of view of querying the oracle, one can reduce all considerations involving the controlled transformation to those involving the phase kick-back transformation, which shall be denoted $\mathcal {O}_f$. (This justifies our use of phase transformations as oracles in Definition 2.2 below.)

As was shown in Sect. 1, all states in theories satisfying our principles can be decomposed as $|s)=\sum _I |s_I)$, with $I\subseteq \{1,\ldots , n\}$, where $\{1,\ldots , n\}$ labels the set of pure and perfectly distinguishable states defining the action of a give oracle. Hence, oracles can not only be queried using a set of pure and perfectly distinguishable states, but also using generalised superposition states—those with non-trivial coherences between different subsets of slits. In fact, the quantum speed-up in the above example came precisely from the fact that one can query in superposition, hence extracting the value $f(0)\oplus f(1)$ in a single query. To ensure that answers to hard to solve problems are not smuggled into the definition of oracles in generalised theories, we must put conditions on which phase transformations correspond to ‘reasonable’ oracles.

We remark that in some definitions of oracle, the possibility of a *null query* is included. This is an input to the oracle transformation conditional on which nothing happens to the target register. Although we did not explicitly include the possibility above, we could define an oracle for $f: S \rightarrow T$ with the possibility of a null query, as above but with an additional distinguishable state of the control register, indexed by some symbol (say $\bullet$) not in *S*, and with $T_{\bullet } = \mathrm {id}$, the identity transformation. Our results still hold for this notion of oracle (which can be viewed as a special case of an oracle of the type defined above, for the slight extension of the function *f* to have one more input, on which it takes a fixed value).

### Definition 2.2

(*Oracle system*) A *system of oracles* for a family $\mathcal {C}$ of functions $S \rightarrow T$ is defined as a family of phase transformations (which we call “oracles” because they are a particular case of the oracles of Definition 2.1) $\{\mathcal {O}_f\}_{f \in \mathcal {C}}$ such that whenever $f(i)=g(i)$ for all $i\in I$, the oracles corresponding to *f* and *g* satisfy

$$\begin{aligned} \mathcal {O}_f|s_I)=\mathcal {O}_g|s_I) \end{aligned}$$

for all $$|s_I)$$ of the form $$\omega _I |s)$$ (for arbitrary |*s*).

An equivalent, and perhaps more intuitively motivated, definition substitutes the condition “ for all states $$|s_I)$$ such that $$|s_I) = P_I |s_I)$$, for the condition “for all $$|s_I)$$ of the form $$|s_I) = \omega _I|s)$$...” This ensures one cannot learn about the value *f*(*j*) when querying using a state with no probability of being found in |*j*). That is, $$\mathcal {O}_f$$ and $$\mathcal {O}_g$$ act identically on states in the face determined by a subset of inputs on which *f* and *g* have the same value, so that we cannot, for example, just write *arbitrary* information about which function is being queried into phase degrees of freedom.

One can schematically represent the problems that can be solved by a specific computational model with access to an oracle using the language of complexity classes. Let $\mathbf {C}$ and $\mathbf {B}$ be complexity classes, then $\mathbf {C}^{\mathbf {B}}$ denotes the class $\mathbf {C}$ with an oracle for $\mathbf {B}$ (see [43] for formal definitions). We can think of $\mathbf {C}^{\mathbf {B}}$ as the class of languages decided by a computation which is subject to the restrictions and acceptance criteria of $\mathbf {C}$, but allowing an extra kind of computational step: an oracle for any desired language $\mathcal {L}\in \mathbf {B}$ that may be queried during the computation, where each query counts as a single computational step. Here $\mathcal {L}$ is fixed in any given computation, though different computations may use different $\mathcal {L}$.

A natural question is whether or not having access to an oracle for a particular decision problem which can be efficiently solved in a given theory provides any more computational power than just using the efficient algorithm. If we schematically denote the class of problems efficiently solvable by a particular theory **G** by^10^ **BGP**, this question can be phrased as: “is **BGP** closed under *subroutines*”? Here **BGP** is the analogue of the well-known class of problems efficiently solvable by a quantum computer, **BQP**. Another way to pose this question is to ask whether $\mathbf BGP ^\mathbf BGP =\mathbf BGP$ for **G** satisfying our principles.

There exist complexity classes for which this is probably not the case, for example, **NP**^11^. But, intuitively, one would expect it to hold in a sensible physical theory where computation is performed with circuits. In such a situation, one might consider it a kind of conceptual consistency check on one’s definition of oracle: it is not reasonable for an oracle for a problem to give more power than would be afforded by a circuit for that problem in the model. A potential issue arises when one compares the performance of the oracle implementation to that of the efficient algorithm when both are used as subroutines in another computational algorithm.^12^ As we noted in Sects. 1 and 2, an oracle can be queried on a superposition of inputs, but one does not normally query an algorithm for a particular decision problem in superposition for the purpose of solving that decision problem; one merely prepares the state corresponding to a particular bit string and uses the algorithm to determine whether or not that bit string is in the language in question.^13^ For simplicity, we say the efficient algorithm accepts an input if a measurement of the first outcome system yields outcome (0| with probability^14^ greater than 2 / 3. This is the same acceptance condition imposed in quantum computation.

We therefore need to know whether every **BGP** algorithm for a decision problem admits a subroutine having the characteristics of an oracle for that decision problem. Such a result was proved in the quantum case by Bennett et al. in [17]. The following theorem shows that it is also true for theories satisfying our principles. (See [3] for the definition of circuit and circuit family in the GPT context.)

### Theorem 2.3

Consider a theory **G** which satisfies the principles outlined in Sect. 1. Given an algorithm (poly-size family of circuits in $\mathbf G$), $\{A_{|x|}\}$, for a decision problem in **BGP**, one can always construct a family $\{G_{|x|}\}$ of polynomial-size circuits implementing reversible transformations from **G**, which, with high probability (greater than $1 - 2^{-q(|x|)}$, for some polynomial *q*), functions as an oracle for that particular decision problem (in the sense of Definition 2.1). (Here, poly-size means polynomial in the length |*x*| of the input *x*, and the family $G_{|x|}$ comprises a fixed circuit for each input size.) Schematically, we have $\mathbf BGP ^\mathbf BGP =\mathbf BGP$.

### Proof

See Appendix C$$\square $$

Given our definition of an oracle we can consider how their computational power depends on the order of interference of the theory.

## Lower Bounds from Useless Queries

In this section we generalise results of Reference [18], in which Meyer and Pommersheim derived a relation between quantum and classical query complexity lower bounds, by introducing the concept of a “useless” quantum query to the setting of GPTs satisfying our principles. They considered *learning problems* in which one is given an element from a class of functions with the same domain and range, chosen with some arbitrary—but known—prior distribution, where the task is to determine to which specific subclass the chosen function belongs.

More formally we can define a learning problem as follows:

### Definition 3.1

(*Learning problems*) Given a set of functions $\mathcal {C}\subseteq \{0,1\}^X$ where *X* is some finite set,^15^ a partitioning of $\mathcal {C}$ into disjoint subsets $\mathcal {C}=\bigsqcup _{j\in J}\mathcal {C}_j$ labeled by $j\in J$, and a probability measure $\mu$ over $\mathcal {C}$. The aim of the learning problem is to determine, which partition $\mathcal {C}_j$ a particular function $f\in \mathcal {C}$ belongs to; this is to be done with low *ex ante* probability of error, with respect to the probability measure $\mu$ with which the function is chosen from among the $\mathcal {C}_j$’s. A particular learning problem is therefore defined by the triple, $(\mathcal {C},\{\mathcal {C}_j\},\mu )$.

One can only access information about the function by querying an oracle, which, when presented with an element from the domain, outputs the corresponding element of the range assigned by the chosen function. Typically one specifies some upper bound to the error probability, and is interested in the minimal number of queries needed to ensure that the *ex ante* probability of error is below the bound, with respect to the measure $\mu$ that gives the “prior probability” of functions $f \in \mathcal {C}$.

Meyer and Pommersheim showed that if *n* queries to a classical oracle reveal no information about which function was chosen^16^ then neither do *n* / 2 queries to a quantum oracle. Hence $\lceil {n/2}\rceil +1$ quantum queries constitute a lower bound.

Many important query problems are examples of learning problems. For instance, PARITY, a generalisation of the special case of Deutsch’s problem where the input to *f* is a bit, [16] which asks for the parity of a function^17^ $f:\{1,\ldots ,N\}\rightarrow \{0,1\}$ can be written as a learning problem. Indeed, partition the class of all such functions into two subclasses, one in which all functions have parity 0 and the other 1, and choose the function with a prior probability of 1 / 2. In this case, $N-1$ classical queries do not provide any information about the parity, hence at least $\lceil {(N-1)/2}\rceil +1$ quantum queries are needed to solve the problem. In fact $\lceil {(N-1)/2}\rceil +1$ quantum queries are also sufficient.^18^

In this section we generalise Meyer and Pommersheim’s result to the case of oracle queries in the generalised probabilistic theory framework presented in the previous section. We prove that if *n* queries to a classical oracle reveal no information about which function was chosen then neither do *n* / *k* queries in a generalised theory satisfying the principles introduced in Sect. 1 and which has maximal order of interference *k*. Hence a lower bound to determining the function is $\lceil {n/k}\rceil +1$ queries in theories with *k*th order interference. So in the specific generalisation of Deutsch’s problem where we are asked to determine the parity of a function $f:\{1,\ldots ,n\}\rightarrow \{0,1\}$, $\lceil {n/2}\rceil$ quantum queries are needed, but in a theory with *n*th order interference, the “ no-information” or “useless-queries” bound does not rule out the possibility that the parity can be determined with a single query.

We can now formally define what it means for *n* classical queries to be useless [18].

### Definition 3.2

(*Useless classical queries* [18]) Let $\left( \mathcal {C}, \{\mathcal {C}_j : j\in J\}, \mu \right)$ be a learning problem. *n* classical queries are said to be *useless*, or to convey no information, if for any $x_1, \ldots x_n\in X$ and $y_1,\ldots y_n \in \{0,1\}$ the following holds

$$\begin{aligned} \mu \left( f\in \mathcal {C}_j \text { } |\text { } f(x_i)=y_i, i=1,\dots ,n\right) =\mu \left( f\in \mathcal {C}_j\right) , \text { for all } j\in J. \end{aligned}$$

Here expressions like $\mu \left( f\in \mathcal {C}_j \text { } |\text { } f(x_i)=y_i, i=1,\ldots ,n\right)$ are to be understood simply as conditional probabilities of events like $f \in \mathcal {C}_j$, conditional on events like $f(x_1)= y_1~ \& ~f(x_2) = y_2 ~ \& ~ \cdots ~ \& ~ f(x_n) = y_n$.

A general *n*-query algorithm in a generalised theory satisfying our principles corresponds to the following: an arbitrary initial state $|\sigma )$ is prepared and input to the oracle $\mathcal {O}_f$, the output state is acted upon by an arbitrary transformation $G_1$ independent of *f*, and the process is repeated. After the *n*th oracle query, the state is

$$\begin{aligned} |\rho _f)=G_n\mathcal {O}_f G_{n-1}\cdots G_1\mathcal {O}_f|\sigma ). \end{aligned}$$

The final step consists of measuring this state with an arbitrary measurement denoted as $\{(s|\}_{s\in S}$. The final^19^ output of the algorithm is given by a map, which is independent of *f* from the set *S* indexing the measurement outcome to the set *J* indexing the subclasses to which the function could belong.

The probability of outcome (*s*| being observed in the measurement, when the unknown function is *f*, is therefore defined to be $(s|\rho _f)$. So there is a joint probability distribution, which we will denote also by the letter $\mu$, over the outcome *s* and the function *f*:

$$\begin{aligned} \mu (s,f) = (s|\rho _f)\mu (f). \end{aligned}$$

Bayes’ rule gives the posterior probability that the function was *f*, given the observed measurement outcome *s*:

$$\begin{aligned} \mu (f|s) = (s|\rho _f)\mu (f) / \sum _{g \in \mathcal {C}} (s|\rho _g)\mu (g). \end{aligned}$$

The posterior probability that $f \in \mathcal {C}_j$ given this outcome is

$$\begin{aligned} \mu (f \in \mathcal {C}_j|s) = \sum _{f in \mathcal {C}_j} \mu (f|s). \end{aligned}$$

Similarly we define $\mu (f \in C_j) = \sum _{f \in \mathcal {C}_j} \mu (f)$, the prior probability that $f \in C_j$.

We can now generalise the definition of a useless quantum query from [18] to the case of generalised theories satisfying our principles.

### Definition 3.3

(*Useless generalised queries*) Let $\left( \mathcal {C}, \{\mathcal {C}_j \}_{j\in J}, \mu \right)$ be a learning problem. *n* generalised queries are said to be *useless*, or to convey no information, if for any *n* query generalised algorithm with initial state $|\sigma )$, transformations $G_n,\dots , G_1$, and measurement $\{(s|\}_{s\in S}$ the following holds

$$\begin{aligned} \mu \left( f\in \mathcal {C}_j \text { } |\text { } s\right) = \mu \left( f\in \mathcal {C}_j\right) , \text { for all possible } s\in S, j\in J. \end{aligned}$$

We now present our main result, which generalises Theorem 1 from [18].

### Theorem 3.4

Let $\left( \mathcal {C}, \{\mathcal {C}_j : j\in J\}, \mu \right)$ be a learning problem. Suppose *kn* classical queries are useless. Then in any theory which satisfies our principles and has maximal order of interference *k*, *n* generalised queries are useless.

We present the formal proof below, but first provide a rough sketch proof. In a theory with *k*th order interference, each state can be decomposed as in Eq. (1.3). Hence each state is explicitly indexed by subsets—of size at most $|I|=k$—of the set of pure and perfectly distinguished states defining the oracle. Thus, after a single generalised query, the state is indexed by the valuation of the chosen function on at most *k* inputs. After *n* generalised queries, it is indexed by *kn* valuations. Therefore, a measurement can reveal at most *kn* valuations of the chosen function. But, as *kn* classical queries are useless, it must be that *n* generalised queries are also useless. The intuition behind this result is that, as a given state can have coherence between at most *k* basis states, one can use generalised superposition states to extract at most *k* valuations of a given function in a single query.

### Proof

Our proof is essentially a slight generalisation of the original quantum one presented in [18]. We need to show that the probability of *f* being in $\mathcal {C}_j$ does not change if outcome *s* is observed after *n* queries. That is, we must show

$$\begin{aligned} \sum _{f\in \mathcal {C}_j} \mu (f\text { }|\text { } s) = \mu \left( \mathcal {C}_j\right) , \text { for any } s\in S \text { and } j\in J. \end{aligned}$$

Recalling from the application of Bayes’ rule to obtain Eq. 3 we have:

$$\begin{aligned} \mu (f|s)= \frac{(s|\rho _f)\mu (f)}{\sum _{g\in \mathcal {C}} (s|\rho _g)\mu (g)} \end{aligned}$$

and summing over *f* in $\mathcal {C}_j$, we have

3.1 $$\begin{aligned} \sum _{f\in \mathcal {C}_j}\mu (f\text { }|\text { } s) = \frac{\left( s\left| \sum _{f\in \mathcal {C}_j} \mu (f)\right| \rho _f\right) }{\left( s\left| \sum _{g\in \mathcal {C}} \mu (g)\right| \rho _g\right) }. \end{aligned}$$

Let’s focus on $|\rho _f)$. Given the decomposition in Eq. (1.3), every state can be written as

$$\begin{aligned} |\sigma )=\sum _{I,|I|=1}^k\omega _I|\sigma )=: \sum _{I,|I|=1}^k\sigma _I. \end{aligned}$$

Now, each $\mathcal {O}_f\left( \sigma _I\right)$ can depend on all *f*(*i*) with $i\in I$. By padding out those *I* with $|I|<k$ with dummy indices, after a single query one can write

$$\begin{aligned} \mathcal {O}_f |\sigma )=\sum _I \mathcal {O}_f(\sigma _I)=\sum _{T_1} Q_{T_1}\left( f(x_1^1), f(x^2_1),\dots , f(x^k_1)\right) , \end{aligned}$$

where the second equality is just a relabeling of the terms where $T_1=\{x_1^1, x^2_1,\ldots , x^k_1\}$ is the padded version of *I*, and hence each $Q_{T_1}$ is a vector in the real vector space of states that depends on $f(x_1^1), f(x^2_1),\ldots , f(x^k_1)$. Therefore, after *n* queries one can write the state as

$$\begin{aligned} |\rho _f)= \sum _{T_n} Q_{T_n}\left( f(x_1^1),\dots , f(x^1_n), f(x^2_1),\ldots , f(x^2_n),\dots , f(x^k_1),\ldots ,f(x^k_n) \right) \end{aligned}$$

Using a change of variables provides

$$\begin{aligned} \begin{aligned}&\sum _{f\in \mathcal {C}_j} \mu (f)|\rho _f) \\&\quad =\sum _{T_n}\sum _{\{y_i^1\},\ldots ,\{y^k_i\}} \mu \left( f\in \mathcal {C}_j \text { and } f(x_i^m)=y_i^m, \text { for } i=1,\ldots ,n \text { and } m=1,\dots , k\right) \\&\qquad \times Q_{T_n}\left( y_1^1,\ldots y_n^k \right) . \end{aligned} \end{aligned}$$

As *kn* classical queries are useless

$$\begin{aligned} \begin{aligned}&\mu \left( f\in \mathcal {C}_j \text { and } f(x_i^m)=y_i^m, \text { for } i=1,\ldots ,n \text { and } m=1,\ldots , k\right) \\&\quad = \mu (\mathcal {C}_j) \mu \left( f(x_i^j)=y_i^j, \text { for } i=1,\ldots ,n \text { and } j=1,\ldots , k\right) . \end{aligned} \end{aligned}$$

Inputting this into the above we obtain,

3.2 $$\begin{aligned} \begin{aligned}&\sum _{f\in \mathcal {C}_j} \mu (f)|\rho _f) \\&\quad = \mu (\mathcal {C}_j) \sum _{T_n}\sum _{\{y_i^1\},\ldots ,\{y^k_i\}}\mu \left( f(x_i^j)=y_i^j, \text { for } i=1,\ldots ,n \text { and } j=1,\ldots , k\right) \\&\qquad \times Q_{T_n}\left( y_1^1,\ldots y_n^k \right) . \end{aligned} \end{aligned}$$

Then. summing over $j\in J$, results in

$$\begin{aligned} \begin{aligned}&\sum _{f\in \mathcal {C}} \mu (f)|\rho _f) \\&\quad =\sum _{T_n}\sum _{\{y_i^1\},\dots ,\{y^k_i\}}\mu \left( f(x_i^j)=y_i^j, \text { for } i=1,\ldots ,n \text { and } j=1,\ldots , k\right) \\&\qquad \times Q_{T_n}\left( y_1^1,\ldots y_n^k \right) . \end{aligned} \end{aligned}$$

Substituting this back into Eq. (3.2) immediately gives

$$\begin{aligned} \sum _{f\in \mathcal {C}_j} \mu (f)|\rho _f) = \mu (\mathcal {C}_j)\sum _{f\in \mathcal {C}}\mu (f)|\rho _f). \end{aligned}$$

finally, substituting this into Eq. (3.1) completes the proof. $\square$

## Conclusion

In this work we have introduced a well-defined oracle model for generalised probabilistic theories, and shown it to be well-behaved in the sense given by our subroutine theorem: that an oracle of our type for a given problem is not more powerful than an algorithm for that problem, since an algorithm permits high-probability simulation of an oracle. This allowed us to compare the computational power imposed by different physical principles through the lens of query complexity. Our main result in this regard was to show that the “zero-information” lower bound on the number of queries to a quantum oracle needed to solve certain problems is not optimal in the space of generalised theories satisfying the principles introduced in Sect. 1. Our result highlights the role of interference in computational advantages in a theory independent manner, allowing the possibility that “more interference could permit more computational power”.

Previous work by the authors in [5] derived Grover’s lower bound to the search problem from simple physical principles. The search problem asks one to find a certain “marked item” from among a collection of items in an unordered database. The only access to the database is through an oracle; when asked if item *i* is the marked one, the oracle outputs “yes” or “no”. The figure of merit in this problem is how the minimum number of queries required to find the marked item scales with the size of the database. It was shown—subject to strong assumptions close to those used in the present paper—that, asymptotically, higher-order interference does not provide an advantage over quantum theory in this case. As opposed to the asymptotic behaviour of the number of queries needed to solve the search problem, the current work was concerned with whether a fixed number of queries yielded any information about the solution of a particular query problem, where the problem could be any of a large class of “learning problems”. In this case we were able to show that the “useless-queries” or “zero-information” lower bound on the number of queries is lower, the higher the order of interference in the theory, leaving open the possibility that higher-order interference could lead to a computational speedup (although we did not show that such a speedup is achievable). Note that a specific oracle model for the search problem was introduced in [5]. However, this is just a special case of the general model introduced in Sect. 2 of the current work. Moreover, the subroutine theorem proved here shows that our general oracle model is well-defined.

Our derivation of query lower bounds raises the question of whether the physical principles we have discussed are sufficient for the existence of algorithms which achieve these lower bounds. In the specific case of the search problem, a quantum search algorithm based on Hamiltonian simulation, due to Farhi and Gutmann [44] and also presented in chapter 6 of the well known textbook by Nielsen and Chuang [16], may be more directly generalisable to theories satisfying our principles than Grover’s original construction [45]. This approach may also be applicable to many other query algorithms. In the algorithm as presented in [16] they consider a Hamiltonian *H* consisting of projectors onto the marked item $|x\rangle$ and the initial input state $|\psi \rangle =\alpha |x\rangle +\beta |y\rangle$, with $|y\rangle$ orthogonal to $|x\rangle$ and $\alpha ^2 +\beta ^2=1$, respectively. That is, they consider the Hamiltonian $H=|x\rangle \langle x|+|\psi \rangle \langle \psi |$. Evolving the initial input state under this Hamiltonian for time *t* results in

$$\begin{aligned} \exp (-itH)|\psi \rangle =\cos (\alpha t)|\psi \rangle - i\sin (\alpha t)|x\rangle . \end{aligned}$$

Hence, measuring the system in the $\{|x\rangle , |y\rangle \}$ basis at time $t=\pi /2\alpha$ yields outcome $|x\rangle$ with probability one. If the initial state was a uniform superposition over the orthonormal basis containing $|x\rangle$, then the required evolution time is $t=\pi \sqrt{N}/2$, where *N* is the size of the system (or equivalently, the number of elements in the database being searched).

One might wonder why there is no mention of an oracle in the above discussion. The oracle comes into play when constructing a quantum circuit to simulate the above Hamiltonian evolution. As the above Hamiltonian depends on the marked item, the quantum circuit simulating it must query the search oracle a number of times proportional to the evolution time [16]. In this specific case, an efficient Hamiltonian simulation requires $O(\sqrt{N})$ queries to the oracle, yielding an optimal quantum algorithm (up to constant factors) for the search problem. Recently, the authors of [36] have introduced a physical principle, termed “energy observability”, which implies the existence of a continuous reversible time evolution and ensures that the generator of any such evolution—a generalised “Hamiltonian”—is associated to an appropriate observable, which is a conserved quantity under the evolution—the generalised “energy” of the evolving system. Recall from Sect. 1 that the principles we have discussed were sufficient to ensure that projectors onto arbitrary states correspond to allowed transformations. Hence, our previous principles, together with energy observability as introduced in [36], might be sufficient to run the above quantum search algorithm, hence providing a theory independent description of an optimal (up to constant factors) search algorithm. Similar constructions based on Hamiltonian simulation might also show that theories satisfying the above physical principles can reach the query lower bounds derived in this paper.

## Proof of Theorem 1.9

This is an adaptation of the proof of theorem 8 from [36]. Consider a self-dual cone $\mathbf {C}$ with self-dualising inner product $\left<\cdot ,\cdot \right>$. Now consider a set of pure and perfectly distinguishable states $\phi _i$ which are distinguished by the effects $e_i$ such that $(e_i|\phi _j)=\delta _{ij}$. We can define a face *F* of $\mathbf {C}$ as the minimal face generated by the set of states $\{\phi _i\}$, we can moreover define the dual face $F^*:=\left\{ x\in \mathbf {C}\ | \left<x,s\right> \ge 0 \ \ \forall s\in F\right\}$. Appendix A in [36] shows that if $F=F^*$ then there exists a positive projector onto the face *F*.

Consider some $t\in F$. Self-duality of $\mathbf {C}$ implies that $\left<t,s\right>\ge 0 \ \forall s\in F$ hence, $t\in F^*$ and so $F\subseteq F^*$. We therefore just need to prove the converse inclusion and we are done.

To prove this, consider a normalised extremal $x\in F^*$, there must be some $s\in F$ such that $\left<s,x\right>=0$, where moreover, if $\left<s,y\right>=0$ then $y\propto x$. Next we prove two simple results:

(i) *s* is not internal to *F*—assume, for the sake of contradiction, that *s* is internal. Then $\left<x,t\right>=0 \ \forall t\in F$ so $x=0$ and hence is not normalised.
(ii) There exists $t\in F$ such that *s* and *t* are perfectly distinguishable—assume, again to reach a contradiction, that there is no such *t*. This means that given any pure and perfectly distinguishing measurement $\{\epsilon _i\}$ that $(\epsilon _i|s)>0$ for all *i*. Due to strong symmetry, any pure effect appears in such a measurement, therefore $(e|s)>0$ for all pure effects (*e*|, this suffices for tomography hence |*s*) is an internal state, in contradiction with (i).

Theorem 1 from [37] implies that if *s* and *t* are perfectly distinguishable states then $\left<s,t\right>=0$, therefore we know that $t\propto x$ and so $x\in F$. This is true for all extremal normalised $x\in F^*$ it therefore follows from convexity that this is true for all $x\in F^*$ and so we have $F^*\subseteq F$ which concludes the proof.

Hence, projectors $P_F$ onto *F* are positive transformations. It was shown in [23] that in any theory satisfying causality, purification and informationally consistent composition, mathematically well-defined transformations are physical, i.e. they are allowed in the theory. Hence projectors $P_F$ are physically allowed transformations. Moreover, given two faces, *F* and *G*, generated by different subsets of the same pure and perfectly distinguishable set of states, one has $P_FP_G=P_{F\cap G}$.

## Useful Consequences of Our Principles

### Uniqueness of Distinguishing Measurement

Strong symmetry (together with the no restriction hypothesis, which says that all mathematically well-defined measurements are physical) implies that, given any set of pure and perfectly distinguishable states $\{|i)\}$, there exists a unique measurement $\{(j|\}$ such that,

$$\begin{aligned} (i|j)=\delta _{ij}. \end{aligned}$$

See [36, 37] for details. Moreover, for every set $\{(e_j|\}$ such that $(e_j|i)=\alpha _j \delta _{ij}$, it holds that

$$\begin{aligned} (e_j|=\alpha _j(j|. \end{aligned}$$

### Purifications of Completely Mixed States are Dynamically Faithful

As mentioned in Sect. 1, purification implies that there exist *completely mixed* states. Purification implies that there exists a state $|\psi )$ that purifies such a completely mixed state:

This is unique up to reversible transformation. We denote a particular choice of this purification as,

Purifications of completely mixed states are called *dynamically faithful* states [23] and, due to the constraints on parallel composition imposed in section 1, must satisfy the following important condition [23]:

As a special case, of course the purification of the *maximally mixed* state is dynamically faithful. In our applications, however, any dynamically faithful state, purifying some completely mixed state, will do.

## Proof of Theorem 2.3

### Proof

It was shown in [23] that the purification principle implies the ability to dilate any transformation to a reversible one. We use this fact in the construction of the circuit $\{G_{|x|}\}$. Our construction is equivalent to the one employed in the quantum case by [17].

In the construction, each $G_{|x|}$ is given by:

where $U_{|x|}$ is the reversible transformation which dilates^20^ the **BGP** algorithm $A_{|x|}$

and *C* is “controlled bit-flip”, or “generalised CNOT”: a reversible controlled transformation with the lower system as the control

with $|i)\in \{|0), |1)\}$, $T_0=\mathbb {I}$, and where $T_1$ acts as $T_1|i)=|i\oplus 1)$.

To show that the family $G_{|x|}$ functions as an oracle with high probability, thereby proving theorem 2.3, we will show that the probability corresponding to the following closed circuit

is greater than or equal to $1-2^{-q(|x|)}$, for some polynomial *q*(|*x*|), when the algorithm $A_{|x|}$ accepts^21^ the input *x*.

We choose the dynamically faithful state to satisfy

where $p_i\in (0,1]$ and $\sum _i p_i=1$, which can always be achieved without loss of generality (see theorem 6 and corollary 9 from [23]).

We first show that *C* satisfies

C.1

To see this note that uniqueness of measurement (both of the following states give probability $p_0$ for (0|(0|, and probability zero for each of (0|(1|, (1|(0|,  and (1|(1|) implies

From our choice of dynamically faithful state, it then follows that

Dynamical faithfulness then gives Eq. (C.1).

Secondly, we write

where $|\sigma )$ is a normalised state and $\alpha \in (0,1]$. Our choice of acceptance condition, together with the fact that *U* is a dilation of the algorithm *A*, results in

C.2

Combining (C.1) and (C.2) gives

where the last line follows from self-duality. By amplifying the acceptance probability of the original algorithm *A* (see [3] for an in depth discussion of bounded error efficient computation and amplifying acceptance probabilities), we can ensure that when *x* is in the language we have $P_x(acc)\ge 1-2^{-p(|x|)}$ for an arbitrary polynomial *p*(|*x*|). Hence it follows that $P_x(acc)^2\ge 1-2^{-p(|x|)+1}$. If $(\sigma |\sigma )=1$, choosing $p(|x|)=q(|x|)+1$ completes the proof.

The case $(\sigma |\sigma ) < 1$ can be easily dealt with. As $|\sigma )$ and $(\sigma |$ can be efficiently prepared by a poly-size circuit, the factor $(\sigma | \sigma )$ can be approximated by a rational number to high accuracy (this is a consequence of the computational uniformity condition required to define computation in arbitrary physical theories, including quantum theory. See [3] and [4] for an expanded discussion of this point). Hence one can write $(\sigma |\sigma )=1-c2^{-w(|x|)}$, for *w* a polynomial in the size of the circuit and *c* a constant natural number. One can always find a polynomial *q* such that $\left( 1-c2^{-w(|x|)}\right) \left( 1-2^{-p(|x|)+1}\right) \ge 1-2^{-q(|x|)}$. This completes the proof. $\square$

## Footnotes

1 With the exception of [6], which constructs a theory capable of post-quantum computation. However, whether this computational advantage is directly tied to a simple physical principle remains unclear.

2 Operationally this can be seen as saying that one does not need to perform an infinite number of distinct experiments to characterise states

3 The process $\{\mathcal {U}_j\}_{j\in {Y}}$, where *j* index the positions of the classical pointer, is a coarse-graining of the process $\{\mathcal {E}_i\}_{i\in {X}}$ if there is a disjoint partition $\{X_j\}_{j\in {Y}}$ of *X* such that $\mathcal {U}_j=\sum _{i\in {X_j}}\mathcal {E}_i$.

4 or, more accurately, the real vector corresponding to the state.

5 In the following sense: a state that is not pure can be written as a convex combination of other states, which can be thought of as a lack of information about which of these alternative states describes the system; of course in general in a nonclassical theory there are many, incompatible, such convex decompositions of the state. But a pure state admits no such decomposition.

6 Some may prefer another way of glossing this principle: that the capacity of a pair of systems to store classical information should be the same whether they are accessed separately or jointly.

7 A face is a convex set with the property that if $px+(1-p)y$, for some $p\in (0,1)$, is an element then *x* and *y* are also both elements.

8 Other models of quantum oracle queries have been investigated, but this one is by far the most common.

9 There could be many distinct transformations that have the same behaviour on a set of control states. As long as one fixes which transformation corresponds to the oracle, this is not a problem.

10 See references [3, 4, 6] for a rigorous definition of this class.

11 If one assumes that the polynomial hierarchy doesn’t collapse.

12 Here, an algorithm consists of a poly-size uniform circuit. See [3] for the formal definitions.

13 However, a quantum algorithm for one problem can sometimes be used as a subroutine in a quantum algorithm for another problem; in this case, the subroutine sometimes *is* run on a superposition of inputs.

14 This can be amplified to $1-2^{-q}$, where *q* a polynomial in the size of the circuit, by running the circuit in parallel a polynomial number of times. Again, see [3].

15 One could alternatively consider replacing $\{0,1\}$ with a different finite set *Y*. Thus the result proved in this section, and the result proved in [18], also holds in the more general case.

16 That is, if the probability that the chosen function belongs to a given subclass after *n* classical queries is the same as the known prior probability with which it was originally chosen.

17 i.e., the value $$f(1)\oplus \cdots \oplus f(N) \text { mod }2$$

18 $\lceil {(N-1)/2}\rceil +1$ applications of the solution to Deutsch’s problem.

19 As was noted in [18], the final transformation $G_n$ is unnecessary, as it could be incorporated into the measurement.
